# Prevalence and Risk Factors of Speech Delay in Children Less Than Seven Years Old in Saudi Arabia

**DOI:** 10.7759/cureus.48567

**Published:** 2023-11-09

**Authors:** Lena D Alzahrani, Sarah S Aldharman, Ahmed S Almuzaini, Arwa A Aljishi, Nouf M Alrabiah, Fahad H Binshalhoub, Joud A Alhassun, Asmaa S Ghmaird

**Affiliations:** 1 College of Medicine, University of Tabuk, Tabuk, SAU; 2 College of Medicine, King Saud Bin Abdulaziz University for Health Sciences, Riyadh, SAU; 3 College of Medicine, Qassim University, Qassim, SAU; 4 Department of Pediatrics, Maternity and Children Hospital, Al-Ahsa, SAU; 5 College of Medicine, King Faisal University, Al-Ahsa, SAU; 6 College of Medicine, Imam Muhammad Ibn Saud Islamic University, Riyadh, SAU; 7 College of Medicine, Unaizah College of Medicine and Medical Sciences, Qassim University, Qassim, SAU; 8 Department of Pediatrics, University of Tabuk, Tabuk, SAU

**Keywords:** prevalence, children, saudi arabia, language development, speech delay

## Abstract

Introduction

Verbal communication relies on the ability to speak and understand language. Speech is only one part of language; language can also be expressed through gestures, writing, and other nonverbal means. Speech and language disorders are impairments in the ability to produce and comprehend language, including problems with mouth movements and vocalization. There is a scarcity of literature on this topic in Saudi Arabia; therefore, the aim of this study was to assess the prevalence and risk factors of speech delay in children aged less than seven years in Saudi Arabia.

Methods

A cross-sectional self-reported descriptive study was conducted from May 2023 to June 2023 among parents of children less than seven years of age in the Kingdom of Saudi Arabia. Simple convenience sampling was implemented. A structured, self-administered questionnaire was designed and presented to the parents of children less than seven years of age. Categorical data were presented as frequencies and percentages. The analysis included a chi-squared test and a Fisher’s exact test.

Results

A total of 617 participants were included in the study. The majority of children were between 1 and 3 years of age (223, 36.1%) and were male (336, 54.5%). Around 45.5% of the respondents reported that their children may have a speech delay. Children aged >3 to 5 years had a significantly higher prevalence of speech delay (112, 53.1%). Additionally, there was a significant difference in speech delay prevalence between male (170, 50.6%) and female (111, 39.5%, p = 0.006) children. A family history of a developmental communication disorder was significantly associated with speech delay (p < 0.001). Children with speech delay were more likely to have hearing issues (19, 70.4%) and motor issues (19, 70.4%). Moreover, autism spectrum disorder in the child was significantly associated with speech delay (p < 0.001).

Conclusions

The study found that children aged 3 to 5 years had a significantly higher prevalence of speech delay than younger children. There was a significant difference in speech delay prevalence between male and female children. Children with speech delays were more likely to suffer hearing and motor issues. Speech delay was significantly associated with a family history of a developmental communication problem.

## Introduction

Verbal communication depends on the ability to speak and understand language. Speech and language disorders are impairments in the ability to produce and comprehend language, including problems with mouth movements and vocalization [1,2].

When a child is diagnosed with developmental delay, it means they are not meeting developmental milestones at the expected age [1]. Speech refers to the sounds a child makes, whereas language is a measure of their understanding [2]. Children’s speech and language development can be used as a marker for their overall development and intelligence [3]. If a child’s speech is unintelligible or marked by speech sound errors, they may be diagnosed with speech delay [4,5]. There is a strong link between hearing loss and speech delay, but there are other factors that can contribute to speech-language delay, including environmental and biological factors [6,7].

Studies have found that up to 60% of children with speech and language delays do not fully catch up to their peers, and these children are at a higher risk of long-term social, emotional, behavioral, and cognitive problems [8,9]. Speech and language disorders are relatively common among school-aged children, with 3%-20% of children estimated to be experiencing some degree of delay [10]. A study in Pakistan showed that children in primary school have a low (1.3%) prevalence of speech sound disorders, with male children between 5 and 6 years of age having a significantly higher rate of these disorders [11]. Speech and language disorders can significantly affect a child’s development and can be categorized as either primary (no known cause) or secondary (with an established etiology). Hearing difficulties, behavioral or emotional issues, and neurological disorders can all contribute to speech and language delays [12].

There are several risk factors for speech and language disorders in children, including family history, premature birth, low birth weight, male gender, and low socioeconomic status [13]. Otitis media, or middle ear infection, is also a risk factor because it can lead to hearing loss and difficulty with speech and language development [14]. Suckling habits, such as using pacifiers, dummies, thumbs, or bottles, can lead to oral sensory issues and oro-motor dysfunction [15].

Screening all children for speech, language, and hearing difficulties is important because early diagnosis and intervention can prevent long-term consequences such as low IQ, communication difficulties, and illiteracy [15]. Risk factor assessment is vital to developing prevention strategies and improving outcomes for children with speech and language disorders.

The prevalence of speech delay is often overlooked and not considered a cause for alarm [2,7-9]. This leads to late diagnosis and intervention, possibly negatively affecting a child’s development. There is a lack of data on this topic in Saudi Arabia. Therefore, this study was conducted for a better understanding of the prevalence and risk factors of speech delay in children aged less than seven years in Saudi Arabia.

## Materials and methods

Study design

A cross-sectional self-reported descriptive study was conducted. A structured, self-administered questionnaire (Appendix) was designed and presented to parents of children aged less than seven years in Saudi Arabia.

Inclusion and exclusion criteria

All parents of children less than seven years of age in Saudi Arabia were eligible for inclusion. Children older than seven years who refused to participate and children with incomplete data were excluded.

Sample size and sampling method

A convenience sampling technique was implemented. The sample size was estimated with an online sample size calculator (Raosoft, http://www.raosoft.com/samplesize.html) using a margin of error of 5% and a confidence interval (CI) of 95%, assuming an average response rate of 50% for most of the questions. The required sample size was 377 participants.

Data collection tool and technique

The questionnaire was distributed on different online platforms to enable data collection from different regions of Saudi Arabia. The authors thoroughly reviewed the relevant literature before designing the questionnaire. Then it was reviewed by experts in the field of Pediatrics to ensure clarity and simplicity that all items in the questionnaire were relevant for the study purpose. To facilitate reading and comprehension among the populace, the survey was developed in Arabic. Next, utilizing its electronic form, the final questionnaire's content was pretested on 16 individuals with varying demographic features to make sure it was coherent and well-worded. The individuals who took part in the pretest were excluded from the analysis. The questionnaire had six sections. The first section contained questions on the children’s sociodemographic characteristics. The second section contained questions on the parents’ sociodemographic characteristics. The third section contained questions on pregnancy history, including prenatal, natal, and postnatal risk factors. The fourth section contained questions on family and medical history. The fifth section consisted of the speech delay assessment. The last section consisted of the electronic use assessment. Data were collected from May 2023 to June 2023.

Data analysis

Statistical analysis was conducted using RStudio (R version 4.3.0). Categorical data were presented as frequencies and percentages. Multiple response analysis was conducted for variables with multiple choices. Inferential analysis was conducted for assessment of differences between children with and without speech delay in terms of different characteristics. The analysis included a chi-squared test and a Fisher’s exact test. Significantly associated variables were further entered in a logistic regression model to assess independent predictors of speech delay. Statistical significance was considered at p < 0.05.

Ethical considerations

The ethical approval was obtained from the Research Ethics Committee at the University of Tabuk, Saudi Arabia (No. UT-280-126-2023). Participants were informed that their participation was voluntary and optional, with consent form presented before filling the questionnaire. Confidentiality and privacy of participants’ information were safeguarded throughout the study.

## Results

Sociodemographic characteristics

Table 1 shows the sociodemographic characteristics of the children and parents in the study sample (N = 617). The majority of children were between one and three years of age (223, 36.1%) and were male (336, 54.5%). The most common birth order was third or later born (254, 41.2%), and the majority of children were born full term (412, 66.8%). Most households had three or more children (302, 48.9%) with both parents living in the house (584, 94.7%). Urban residence was predominant (552, 89.5%). Regarding childcare, a nanny or someone else did not attend to the majority of children (412, 66.8%), and a majority did not attend nursery (466, 75.5%). Breastfeeding was common among children (390, 63.2%). The mothers were the primary caregivers, with 500 (81.0%) caregivers being mothers. The highest proportion of mothers were in the age group of 25-35 years when they gave birth (390, 63.2%). The highest proportion of fathers were also in the age group of 25-35 years when their wives gave birth (350, 56.7%). Regarding education, the majority of mothers had a university degree or above (420, 68.1%), and the majority of fathers had a university degree or above (404, 65.5%). The majority of fathers were employed (582, 94.3%), and the majority of mothers were housewives (345, 55.9%). The most common average family monthly income was >10,000 Saudi Riyal (277, 44.9%). Regarding the regions of residence, the highest frequency of residence was observed in the southern region (154, 25%).

**Table 1:** Sociodemographic characteristics of the children and parents

Characteristic	Overall, N=617	Speech delay		P-value
		No, N=336	Yes, N=281	
Child age (years)				0.016
1 to 3	223 (36.1%)	135 (60.5%)	88 (39.5%)	
>3 to 5	211 (34.2%)	99 (46.9%)	112 (53.1%)	
>5 to 7	183 (29.7%)	102 (55.7%)	81 (44.3%)	
Child sex				0.006
Male	336 (54.5%)	166 (49.4%)	170 (50.6%)	
Female	281 (45.5%)	170 (60.5%)	111 (39.5%)	
Birth order				0.367
First born	208 (33.7%)	110 (52.9%)	98 (47.1%)	
Second born	155 (25.1%)	92 (59.4%)	63 (40.6%)	
Third or later born	254 (41.2%)	134 (52.8%)	120 (47.2%)	
Gestational age at birth				0.316
Early term	135 (21.9%)	77 (57.0%)	58 (43.0%)	
Full term	412 (66.8%)	227 (55.1%)	185 (44.9%)	
Late term	52 (8.4%)	22 (42.3%)	30 (57.7%)	
Post-term	18 (2.9%)	10 (55.6%)	8 (44.4%)	
Numbers of children at home				0.779
One child	137 (22.2%)	78 (56.9%)	59 (43.1%)	
Two children	178 (28.8%)	97 (54.5%)	81 (45.5%)	
Three or more children	302 (48.9%)	161 (53.3%)	141 (46.7%)	
Living with both parents				0.479
No	33 (5.3%)	16 (48.5%)	17 (51.5%)	
Yes	584 (94.7%)	320 (54.8%)	264 (45.2%)	
Place of residence				0.092
Rural	65 (10.5%)	29 (44.6%)	36 (55.4%)	
Urban	552 (89.5%)	307 (55.6%)	245 (44.4%)	
Nanny or someone else is taking care of the child				0.426
No	412 (66.8%)	229 (55.6%)	183 (44.4%)	
Yes	205 (33.2%)	107 (52.2%)	98 (47.8%)	
Attending nursery				0.122
No	466 (75.5%)	262 (56.2%)	204 (43.8%)	
Yes	151 (24.5%)	74 (49.0%)	77 (51.0%)	
Breastfeeding				0.005
No	227 (36.8%)	107 (47.1%)	120 (52.9%)	
Yes	390 (63.2%)	229 (58.7%)	161 (41.3%)	
Relation to the child				0.791
Father	117 (19.0%)	65 (55.6%)	52 (44.4%)	
Mother	500 (81.0%)	271 (54.2%)	229 (45.8%)	
Age of the mother at birth (years)				0.498
Below 25	106 (17.2%)	63 (59.4%)	43 (40.6%)	
25-35	390 (63.2%)	210 (53.8%)	180 (46.2%)	
More than 35	121 (19.6%)	63 (52.1%)	58 (47.9%)	
Age of the father at birth (years)				0.125
Below 25	24 (3.9%)	14 (58.3%)	10 (41.7%)	
25-35	350 (56.7%)	202 (57.7%)	148 (42.3%)	
More than 35	243 (39.4%)	120 (49.4%)	123 (50.6%)	
Education level of the mother				0.466
Illiterate	10 (1.6%)	7 (70.0%)	3 (30.0%)	
School education	187 (30.3%)	97 (51.9%)	90 (48.1%)	
University degree or above	420 (68.1%)	232 (55.2%)	188 (44.8%)	
Education level of the father				0.045
Illiterate	7 (1.1%)	7 (100.0%)	0 (0.0%)	
School education	206 (33.4%)	112 (54.4%)	94 (45.6%)	
University degree or above	404 (65.5%)	217 (53.7%)	187 (46.3%)	
Occupation of the father				0.015
Employed	582 (94.3%)	310 (53.3%)	272 (46.7%)	
Unemployed	35 (5.7%)	26 (74.3%)	9 (25.7%)	
Occupation of the mother				0.108
Employed	272 (44.1%)	158 (58.1%)	114 (41.9%)	
Housewife	345 (55.9%)	178 (51.6%)	167 (48.4%)	
Family monthly income, Saudi Riyal				0.522
Less than 5,000	64 (10.4%)	39 (60.9%)	25 (39.1%)	
5,000–10,000	276 (44.7%)	150 (54.3%)	126 (45.7%)	
More than 10,000	277 (44.9%)	147 (53.1%)	130 (46.9%)	
Region of residence				0.005
Central region	102 (16.5%)	46 (45.1%)	56 (54.9%)	
Eastern region	127 (20.6%)	84 (66.1%)	43 (33.9%)	
Western region	112 (18.2%)	61 (54.5%)	51 (45.5%)	
Northern region	122 (19.8%)	72 (59.0%)	50 (41.0%)	
Southern region	154 (25.0%)	73 (47.4%)	81 (52.6%)	

Differences in speech delay based on sociodemographic characteristics

**Figure 1:**
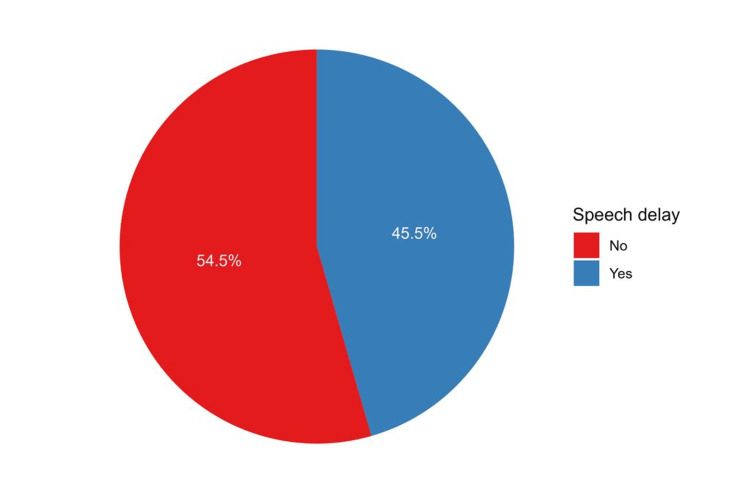
The proportions of participants’ responses regarding their perception of speech delay among their children

In general, less than half of the respondents indicated that their children might have speech delay (n = 281, 45.5%, Figure 1). The inferential analysis results showed that children aged >3 to 5 years had a significantly higher prevalence of speech delay (112, 53.1%) compared to those aged 1 to 3 years (88, 39.5%, p = 0.016). There was a significant difference in speech delay prevalence between male (170, 50.6%) and female (111, 39.5%, p = 0.006) children. Furthermore, children with fathers educated at the university level or above had a high speech delay prevalence (187, 46.3%, p = 0.045). Similarly, children with unemployed fathers had a lower speech delay prevalence (9, 25.7%) compared to those with employed fathers (272, 46.7%). Additionally, speech delay was significantly associated with breastfeeding, with a higher prevalence observed among breastfed children (120, 52.9%) compared to children who were not breastfed (161, 41.3%, p = 0.005). Among the regions of residence, the central region had the highest prevalence of speech delay (56, 54.9%), whereas the eastern region had the lowest (43, 33.9%, p = 0.005, Table 1).

Family history

A family history of developmental communication disorder was strongly associated with speech delay: 42 (72.4%) children with speech delay had at least one immediate family member (parents or siblings) with a developmental communication disorder compared to 190 (39.2%) children with speech delay who had no immediate family member with a developmental communication disorder (p < 0.001). The presence of a hearing issue in any family member was associated with speech delay: 43 (56.6%) children with speech delay had a family member with a hearing issue compared to 238 (44%) children with speech delay who had no family member with a hearing issue (p = 0.039).

Notably, health problems in children were significantly associated with speech delay. Children with speech delay were more likely to have a hearing issue (19, 70.4%) and motor issue (19, 70.4%) compared to children without speech delay (8, 29.6% for both). Regarding autism spectrum disorder (ASD), ASD diagnosis in children was strongly associated with speech delay: 49 (84.5%) children with speech delay had an ASD diagnosis, whereas 232 (41.5%) children with speech delay had no ASD diagnosis (p < 0.001). A family history of ASD was associated with speech delay: 32 (66.7%) children with speech delay had a family member diagnosed with ASD compared to 249 (43.8%) children with speech delay who did not have a family member diagnosed with ASD (p = 0.002, Table 2).

**Table 2:** Family medical history

Characteristic	Overall, N=617	Speech delay		P-value
		No, N=336	Yes, N=281	
A family history of a developmental communication disorder				<0.001
In no immediate family member	485 (78.6%)	295 (60.8%)	190 (39.2%)	
In ≥1 immediate (parents, siblings) family member	58 (9.4%)	16 (27.6%)	42 (72.4%)	
In any non-immediate family members	74 (12.0%)	25 (33.8%)	49 (66.2%)	
Is there any member in the family with a history of hearing issue?				0.039
No	541 (87.7%)	303 (56.0%)	238 (44.0%)	
Yes	76 (12.3%)	33 (43.4%)	43 (56.6%)	
Does your child have any of the following health problems?				
None	531 (86.1%)	305 (57.4%)	226 (42.6%)	<0.001
Hearing issue	27 (4.4%)	8 (29.6%)	19 (70.4%)	0.008
Motor issue	27 (4.4%)	8 (29.6%)	19 (70.4%)	0.008
Visual issue	25 (4.1%)	12 (48.0%)	13 (52.0%)	0.508
Does your child have diagnosed with autism spectrum disorder?				<0.001
No	559 (90.6%)	327 (58.5%)	232 (41.5%)	
Yes	58 (9.4%)	9 (15.5%)	49 (84.5%)	
Do you have any member in your family diagnosed with autism spectrum disorder?				0.002
No	569 (92.2%)	320 (56.2%)	249 (43.8%)	
Yes	48 (7.8%)	16 (33.3%)	32 (66.7%)	

Perinatal factors

Among the perinatal factors shown in Table 3, jaundice was significantly associated with speech delay: 5 (22.7%) children with speech delay had jaundice, whereas 138 (46.5%) children with speech delay did not have jaundice (p = 0.028). No other perinatal factors showed significant associations with speech delay in the study sample.

**Table 3:** Perinatal factors HIE, hypoxic-ischemic encephalopathy

Characteristic	Overall, N=617	Speech delay		P-value
		No, N=336	Yes, N=281	
Consanguinity of parents				0.204
No	329 (53.3%)	187 (56.8%)	142 (43.2%)	
Yes	288 (46.7%)	149 (51.7%)	139 (48.3%)	
Twin/multiple pregnancy				0.359
No	578 (93.7%)	312 (54.0%)	266 (46.0%)	
Yes	39 (6.3%)	24 (61.5%)	15 (38.5%)	
Delivery mode				0.484
Normal vaginal delivery	421 (68.2%)	236 (56.1%)	185 (43.9%)	
Elective caesarian section	57 (9.2%)	30 (52.6%)	27 (47.4%)	
Mandatory cesarean section	139 (22.5%)	70 (50.4%)	69 (49.6%)	
Antenatal factors - none				0.607
No	164 (26.6%)	92 (56.1%)	72 (43.9%)	
Yes	452 (73.3%)	243 (53.8%)	209 (46.2%)	
Antenatal factors - diabetes				0.561
No	552 (89.5%)	298 (54.0%)	254 (46.0%)	
Yes	64 (10.4%)	37 (57.8%)	27 (42.2%)	
Antenatal factors - using medications				0.567
No	568 (92.1%)	307 (54.0%)	261 (46.0%)	
Yes	48 (7.8%)	28 (58.3%)	20 (41.7%)	
Antenatal factors - hypertensive disorder				0.353
No	570 (92.4%)	313 (54.9%)	257 (45.1%)	
Yes	46 (7.5%)	22 (47.8%)	24 (52.2%)	
Antenatal factors - hypoxia				0.456
No	615 (99.7%)	335 (54.5%)	280 (45.5%)	
Yes	1 (0.2%)	0 (0.0%)	1 (100.0%)	
Antenatal factors - others				0.389
No	590 (95.6%)	323 (54.7%)	267 (45.3%)	
Yes	26 (4.2%)	12 (46.2%)	14 (53.8%)	
Intrapartum factor - did not cry immediately				0.978
No	582 (94.3%)	316 (54.3%)	266 (45.7%)	
Yes	33 (5.3%)	18 (54.5%)	15 (45.5%)	
Intrapartum factor - premature				0.894
No	588 (95.3%)	319 (54.3%)	269 (45.7%)	
Yes	27 (4.4%)	15 (55.6%)	12 (44.4%)	
Intrapartum factor - fetal distress				0.119
No	577 (93.5%)	318 (55.1%)	259 (44.9%)	
Yes	38 (6.2%)	16 (42.1%)	22 (57.9%)	
Intrapartum factor - hypoxia				0.128
No	561 (90.9%)	310 (55.3%)	251 (44.7%)	
Yes	54 (8.8%)	24 (44.4%)	30 (55.6%)	
Intrapartum factor - others				0.830
No	601 (97.4%)	326 (54.2%)	275 (45.8%)	
Yes	14 (2.3%)	8 (57.1%)	6 (42.9%)	
Postnatal factors - none				0.952
No	130 (21.1%)	71 (54.6%)	59 (45.4%)	
Yes	486 (78.8%)	264 (54.3%)	222 (45.7%)	
Postnatal factors - admission to the NICU				0.488
No	574 (93.0%)	310 (54.0%)	264 (46.0%)	
Yes	42 (6.8%)	25 (59.5%)	17 (40.5%)	
Postnatal factors - hypoglycemia				>0.999
No	615 (99.7%)	334 (54.3%)	281 (45.7%)	
Yes	1 (0.2%)	1 (100.0%)	0 (0.0%)	
Postnatal factors - cyanosis				0.275
No	597 (96.8%)	327 (54.8%)	270 (45.2%)	
Yes	19 (3.1%)	8 (42.1%)	11 (57.9%)	
Postnatal factors - HIE/neonatal seizures				0.169
No	600 (97.2%)	329 (54.8%)	271 (45.2%)	
Yes	16 (2.6%)	6 (37.5%)	10 (62.5%)	
Postnatal factors - jaundice				0.028
No	594 (96.3%)	318 (53.5%)	276 (46.5%)	
Yes	22 (3.6%)	17 (77.3%)	5 (22.7%)	
Postnatal factors - low birth weight				0.123
No	568 (92.1%)	314 (55.3%)	254 (44.7%)	
Yes	48 (7.8%)	21 (43.8%)	27 (56.3%)	
Postnatal factors - others				0.383
No	611 (99.0%)	331 (54.2%)	280 (45.8%)	
Yes	5 (0.8%)	4 (80.0%)	1 (20.0%)	

Assessment of using electronic devices

In the assessment of electronic device usage among children with speech delay, several variables showed significant associations (p < 0.05). The prevalence of speech delay increased significantly as the hours of TV watching per day increased. Speech delay was reported among 35.4%, 47.3%, and 52.2% of children who watched TV for <1 hour, 1-3 hours, and > 3 hours, respectively (p = 0.005). The prevalence of speech delay was thus significantly higher among those who watched TV (53.5%) than among those who did not watch it (38.1%, p < 0.001). Meanwhile, children who did not use electronic devices had a lower proportion of speech delay compared to children who used electronic devices (36.0% vs. 47.4%, p = 0.036, Table 4).

**Table 4:** Assessment of using electronic devices

Characteristic	Overall, N=617	Speech delay		P-value
		No, N=336	Yes, N=281	
What types of applications does your child use?				0.188
Educational app	89 (14.4%)	50 (56.2%)	39 (43.8%)	
Minecraft	48 (7.8%)	33 (68.8%)	15 (31.3%)	
None	27 (4.4%)	17 (63.0%)	10 (37.0%)	
Others	14 (2.3%)	9 (64.3%)	5 (35.7%)	
TV	6 (1.0%)	2 (33.3%)	4 (66.7%)	
YouTube	432 (70.0%)	225 (52.1%)	207 (47.9%)	
Hour(s) of television watching per day				0.005
<1 hour	164 (26.6%)	106 (64.6%)	58 (35.4%)	
1-3 hours	273 (44.2%)	144 (52.7%)	129 (47.3%)	
>3 hours	180 (29.2%)	86 (47.8%)	94 (52.2%)	
Devices the child spends time using - no devices used				0.036
No	517 (83.8%)	272 (52.6%)	245 (47.4%)	
Yes	100 (16.2%)	64 (64.0%)	36 (36.0%)	
Devices the child spends time using - smartphones				0.149
No	364 (59.0%)	207 (56.9%)	157 (43.1%)	
Yes	253 (41.0%)	129 (51.0%)	124 (49.0%)	
Devices the child spends time using - TV				<0.001
No	320 (51.9%)	198 (61.9%)	122 (38.1%)	
Yes	297 (48.1%)	138 (46.5%)	159 (53.5%)	
Devices the child spends time using - tablets				0.790
No	469 (76.0%)	254 (54.2%)	215 (45.8%)	
Yes	148 (24.0%)	82 (55.4%)	66 (44.6%)	

Speech delay assessment

In the speech delay assessment, significant associations (p < 0.001) were found between speech delay and several characteristics. To elaborate, a higher percentage of children with speech delay had lower clarity of speech. The approximate percentage that the child is understood by others (clarity of their speech to others) is as follows: 45 (68.2%) children were understood by others at a rate of 10%-20%, 97 (70.3%) of children were understood by others at a rate of 20%-50%, and 81 (43.3%) of children were understood by others at a rate of 50%-70%. Regarding language development milestones, children with speech delay demonstrated delayed progress. In particular, 198 (60.7%) children had not yet started telling a story with at least two events, whereas 83 (28.5%) children had started telling a story with at least two events. Similarly, 95 (70.4%) children with speech delay had not yet developed the ability to answer simple questions, whereas 186 (38.6%) children with speech delay had developed that ability (p < 0.001). Moreover, children with speech delay did not have the ability to recognize and use simple rhymes (e.g., bat-cat, ball-tall): 94 (58.8%) children had not yet developed the ability to recognize or use rhymes compared to 187 (40.9%) children without speech delay who had developed this ability (p < 0.001). Finally, the way children expressed themselves significantly differed. A higher proportion of children with speech delay relied on pointing gestures as their primary means of expression (75.9% of children with speech delay vs. 24.1% without), whereas a higher percentage of children without speech delay used three words or more in a sentence as their primary mean of expression (74.3% of children without speech delay vs. 25.7% with, p < 0.001, Table 5).

**Table 5:** Speech delay assessment

Characteristic	Overall, N=617	Speech delay		P-value
		No, N=336	Yes, N=281	
The approximate percentage that the child is understood by others or clarity of their speech				<0.001
10%-20 %	66 (10.7%)	21 (31.8%)	45 (68.2%)	
20%-50%	138 (22.4%)	41 (29.7%)	97 (70.3%)	
50%-70%	187 (30.3%)	106 (56.7%)	81 (43.3%)	
More than 70%	226 (36.6%)	168 (74.3%)	58 (25.7%)	
Does your child tell a story she/he heard or made up with at least two events? For example, a cat was stuck in a tree and a firefighter saved it.				<0.001
Not yet	326 (52.8%)	128 (39.3%)	198 (60.7%)	
Yes	291 (47.2%)	208 (71.5%)	83 (28.5%)	
Does your child answer any simple questions?				<0.001
Not yet	135 (21.9%)	40 (29.6%)	95 (70.4%)	
Yes	482 (78.1%)	296 (61.4%)	186 (38.6%)	
Does your child use or recognize simple rhymes (bat-cat, ball-tall)?				<0.001
Not yet	160 (25.9%)	66 (41.3%)	94 (58.8%)	
Yes	457 (74.1%)	270 (59.1%)	187 (40.9%)	
How does your child express?				<0.001
Pointing (gesture)	108 (17.5%)	26 (24.1%)	82 (75.9%)	
One word	90 (14.6%)	37 (41.1%)	53 (58.9%)	
Two words	112 (18.2%)	45 (40.2%)	67 (59.8%)	
Three words or more in a sentence	307 (49.8%)	228 (74.3%)	79 (25.7%)	

Predictors of speech delay

We included all variables showing significant associations with speech delay in a multivariable binary logistic regression model to assess independent predictors of speech delay. Results showed that male children had a higher odds ratio of 1.54 (95% CI: 1.05 to 2.27, p = 0.026) for speech delay compared to female children. Furthermore, employed fathers had a significantly higher odds ratio of 2.85 (95% CI: 1.15 to 7.80, p = 0.030) for speech delay compared to unemployed fathers. Regarding the region of residence, children living in the eastern region had a significantly lower odds ratio of 0.32 (95% CI: 0.17 to 0.60, p < 0.001) for speech delay compared to those living in the central region. The other regions (western, northern, and southern) did not show significant associations with speech delay. Notably, having a family history of developmental communication disorder in either immediate or non-immediate family members was significantly associated with speech delay. The odds ratio was 4.69 (95% CI: 2.27 to 10.2, p < 0.001) for ≥1 immediate family member and 2.83 (95% CI: 1.52 to 5.38, p = 0.001) for non-immediate family members.

Next, children with a diagnosis of ASD had a significantly higher odds ratio of 17.3 (95% CI: 6.35 to 61.8, p < 0.001) for speech delay compared to children without a diagnosis of ASD. Hours of TV watching per day also predicted speech delay: children watching TV for 1-3 hours per day and > 3 hours per day had higher odds ratios of 2.01 (95% CI: 1.20 to 3.40, p = 0.009) and 2.80 (95% CI: 1.59 to 5.00, p < 0.001), respectively, for speech delay compared to children watching TV for <1 hour per day.

Concerning postnatal factors, children with a history of jaundice had a significantly lower odds ratio of 0.10 (95% CI: 0.02 to 0.40, p = 0.002) for speech delay. Conversely, children with a hearing issue had a significantly higher odds ratio of 5.14 (95% CI: 1.76 to 17.6, p = 0.005) for speech delay (Table 6).

**Table 6:** Results of the multivariable regression analysis for the predictors of speech delay OR, odds ratio; CI, confidence interval

Characteristic	OR	95% CI	P-value
Child age (year)			
1 to 3	Reference	Reference	
>3 to 5	1.43	0.92-2.24	0.111
>5 to 7	0.76	0.47, 1.23	0.271
Child sex			
Female	Reference	Reference	
Male	1.54	1.05-2.27	0.026
Education level of the father			
School education	Reference	Reference	
Illiterate	NA	NA	0.979
University degree or above	0.95	0.64-1.41	0.787
Occupation of the father			
Unemployed	Reference	Reference	
Employed	2.85	1.15-7.80	0.030
Region of residence			
Central region	Reference	Reference	
Eastern region	0.32	0.17-0.60	<0.001
Western region	0.63	0.34-1.18	0.149
Northern region	0.54	0.29-1.00	0.051
Southern region	0.96	0.54-1.70	0.893
A family history of a developmental communication disorder			
In no immediate family member	Reference	Reference	
In ≥1 immediate (parents, siblings) family member	4.69	2.27-10.2	<0.001
In any non-immediate family members	2.83	1.52-5.38	0.001
Is there any member in the family with a history of hearing issue?			
No	Reference	Reference	
Yes	1.26	0.70-2.27	0.439
Does your child have diagnosed with autism spectrum disorder?			
No	Reference	Reference	
Yes	17.3	6.35-61.8	<0.001
Do you have any member in your family diagnosed with autism spectrum disorder?			
No	Reference	Reference	
Yes	0.72	0.30-1.71	0.447
Hour(s) of television watching per day			
<1 hour	Reference	Reference	
1-3 hours	2.01	1.20-3.40	0.009
>3 hours	2.80	1.59-5.00	<0.001
Having a hearing issue	5.14	1.76-17.6	0.005
Having a motor issue	2.65	0.98-7.78	0.062
Postnatal factors - jaundice	0.10	0.02-0.40	0.002
Devices the child spends time using - no devices used	1.00	0.55-1.83	0.994
Devices the child spends time using - TV	1.34	0.88-2.03	0.168

## Discussion

Speech delay, which is defined as a developmental delay in the emergence of spoken language, is a common pediatric condition that can significantly affect a child’s development. Previous studies have reported a prevalence of speech delay ranging from 2.1% to 11.4% among preschool children worldwide [10-12]. However, there are limited data on the prevalence of speech delay among children in Saudi Arabia. The aim of this study was to determine the prevalence of speech delay among children aged less than seven years in Saudi Arabia and to identify the associated risk factors.

The results showed a relatively low prevalence of speech delay among children aged less than seven years in Saudi Arabia. The prevalence of speech delay was 45.5%, which is comparable to the global rates of prevalence of speech and language impairments in children (3%-20%) [10]. Similar findings were observed in research where 33% of male children and 19% of female children had speech and language delays [16]. This difference in prevalence could be due to several factors, such as variations in the definitions of speech delay, the methods used to assess speech delay, and the characteristics of the study population.

It is also possible that the higher prevalence of speech delay in this study was due to cultural or linguistic differences. For example, the concept of speech delay may be interpreted differently in Saudi Arabia than in other countries. Additionally, the lower prevalence may be due to a difference in the prevalence of risk factors for speech delay in Saudi Arabia compared to other countries [13,14].

This study showed that the male gender was a significant risk factor for speech delay. This is consistent with previous studies. Other studies have shown a higher incidence of speech-language delay in male children. This is attributed to the slower maturation of the central nervous system in male children and the influence of testosterone, which has been shown to increase the risk of delay in language development [17-19].

A positive family history of speech-reading disorders (stuttering, unclear speech, late speaking, poor vocabulary, dyslexia), with the affected member being a first-degree relative, has been known to be associated with speech and language delay [20]. Parents with better education not only engage their children more but also use more complex words that stimulate and enhance their children’s language skills [3].

The results also showed that children with fathers educated at the university level or above had a high prevalence of speech delay (187, 46.3%, p = 0.045). Thus, educated parents are more likely to recognize speech issues with their child and seek help. This is in line with previous studies that have shown an association between parental education level and speech delay [16]. Lower parental education level is related to lower socioeconomic status, which in turn is associated with an increased risk of speech delay [13]. Lower socioeconomic status may be related to factors such as limited access to early intervention services, increased stress levels, and exposure to environmental toxins [13]. Maternal participation is widely associated with communication development in children. It includes motivating the child to speak, offering insightful comments, telling stories, and involving the child in reading [15]. The literacy of fathers and mothers also affects the development and speech of a child. In a previous study of parents with children who had delayed speech and language, 22.7% of mothers were illiterate, 46.7% of mothers had primary education, 6.7% of mothers had higher secondary education, and 24% of mothers were graduates [13]. Mondal et al. also indicated that maternal illiteracy is a risk factor for speech and language delay [16].

The current study found that among the perinatal factors, jaundice was significantly associated with speech delay. No other perinatal factors showed significant associations with speech delay. This is different from previous studies that showed a significant association between birth asphyxia and speech delay. This difference could be attributed to the lower sample in the current study of participants who had positive perinatal adverse events [21]. Postnatal factors including seizures were not significantly associated with speech delay, which may be explained by the low number of children with seizure disorder in the current study. This result was also different from that of a previous study showing seizure disorder to be a significant risk factor in speech and language delay [21].

The results of the current study showed that a higher proportion of children with speech delay relied on pointing gestures as their primary means of expression (82, 75.9%). This finding showed an association between word learning difficulties and speech delay. It is possible that difficulties in learning new words may be related to underlying neurological or cognitive deficits affecting speech development [22].

Speech and language delay causes impairment of the intelligence of the child and impairment of the development of the child’s mental capabilities [12,16]; therefore, timely diagnosis is necessary to prevent the long-term effects of such delay. Children who are exposed to any risk factor for speech and language delay should be monitored and taken to a speech therapy clinic for a checkup [23].

The current study has certain limitations. First, we used an online self-administered tool to record responses, which may have caused social desirability and recall bias. Also, there was a difference between response rates from Saudi Arabia regions. The study was limited by being a questionnaire, which inherently has the risk of recall and response bias, and being online, effectively restricting the ability to physically evaluate and examine cases. Additionally, the number of children with positive antenatal, intrapartum, and postnatal factors was small, which may have affected the results. Therefore, a larger sample of children with speech delay would have improved the statistical significance of the results.

## Conclusions

Male gender, an age range of three to five years, parental education level, family history of developmental communication disorder, a history of ASD, increased hours of TV watching per day, and hearing issues were all associated factors with an increased risk of speech delay. These findings suggest that multiple factors may be involved in the development of speech delay. A high proportion of children with speech delay depend on pointing gestures as their primary means of expression. Further research is needed to better understand the complex interplay of factors contributing to speech delay.

## Appendices

Questionnaire

A. Sociodemographic characteristics of the studied children.

1.  Child’s age

-    1 - 3 years

-    3 - 5 years

-    5 - 7 years

2.  Child sex

-    Female

-    Male

3.  Birth order

-    First born

-    Second born

-    Third or later born

4.  Gestational age at birth

-    Early term (37 weeks- 38 weeks + 6 days)

-    Full term (39 weeks- 40 weeks+ 6 days)

-    Late term (41 weeks- 41 weeks+ 6 days)

-    Post-term (42 weeks and beyond)

5.  Number of children at home

-    1 child

-    2 children

-    3 or more children

6.  Living with both parents

-    Yes

-    No

7.  Place of residence

-    Rural

-    Urban

8.  Nanny or someone else is taking care of the child

-    Yes

-    No

9.  If your answer to the question is Yes, is the language of the one who cares about the child the same as the language of the child?

-    Yes

-    No

10.  Attending nursery

-    Yes

-    No

11.  Breastfeeding

-    Yes

-     No

12.  If your answer was Yes in the previous question, what is the duration of breastfeeding

-    Less than 6 months

-    6 to less than 12 months

-    12 to 24 months

B.   Sociodemographic characteristics of the parents

13.  Relation to the child

-    Father

-    Mother

14.  Age of the mother at birth

-    Below 25

-    25-35

-    More than 35

15.  Age of the father at birth

-    Below 25

-    25-35

-    More than 35

16.  Education level of the mother

-    Illiterate (no education)

-    School education (elementary, intermediate, or high school)

-    University degree or above

17.  Education level of the father

-    Illiterate (no education)

-    School education (elementary, intermediate, or high school)

-    University degree or above

18.  Occupation of the father

-    Employed

-    Unemployed

19.  Occupation of the mother

-    Employed

-    Housewife

20.  Family monthly income, Saudi Riyal

-    Less than 5000

-    5000-10000

-    More than 10000

C.  Pregnancy history: prenatal, natal, and postnatal risk factors among studied children

21.  Parents' consanguinity

-    Yes

-    No

22.  Twin/multiple pregnancy

-    Yes

-    No

23.  Delivery mode

-    Normal vaginal delivery

-    Elective caesarian section

-    Mandatory cesarean section

24.  Antenatal factor

-    Hypertensive disorder

-    Diabetes

-    Using medications

-    No antenatal complications

-    Other, what?

25.  Intrapartum factor

-    Fetal distress

-    Hypoxia

-    Did not cry immediately

-    Premature

-    Other, what?

26.  Postnatal factors

-    HIE, hypoxic-ischemic encephalopathy/neonatal seizures

-    Jaundice

-    Cyanosis

-    Low birth weight

-    Admission to the NICU

-    Other, what?

D.  Family and medical history

27.  Is there any member in the family with a history of developmental communication disorder (e.g., e., strutting, ring, developmental articulation disorder, or developmental language disorder such as difficulties in the pronunciation of speech sound in words and in the development of vocabulary and sentence structure)?

-    In ≥1 immediate (parents, siblings) family member

-    In no immediate family member

28.  Is there any member in the family with a history of hearing issue?

-    Yes

-    No

29.  Does your child have any of the following health problems?

-    Hearing issue

-    Visual issue

-    Motor issue

-    Other, what?

-    Doesn’t have

30.  Does your child have autism spectrum disorder?

-    Yes

-    No

31.  Do you have any member in your family diagnosed with autism spectrum disorder?

-    Yes

-    No

E.  Speech delay assessment

32.  Do you think your child has a speech delay?

-    Yes

-    No

33.  What is the approximate percentage that the child is understood by others or clarity of their speech?

-    10%-20 %

-    20%-50%

-    50%-70%

-    More than 70%

34.  Does your child tell a story she/he heard or made up with at least two events? For example, a cat was stuck in a tree and a firefighter saved it.

-    Yes

-    Not yet

35.  Does your child answer any simple questions?

-    Yes

-    Not yet

36.  Does your child use or recognize simple rhymes (bat-cat, ball-tall)?

-    Yes

-    Not yet

37.  How does your child express?

-    Pointing (gesture)

-    One word

-    Two words

-    Three words or more in a sentence

F.  Electronic use assessment

38.  Devices the child spends time using

-    Tablets

-    Smartphones

-    TV

-    No devices used

39.  What types of applications does your child use?

-    YouTube

-    Minicraft

-    Educational application

-    Other, mention?

40.  Hour (s) of television watching per day

-    <1 hour

-    1-3 hours

-    >3 hours
